# Theoretical Exploration of Error Thresholds for Clinical AI Decision Support in Nursing: Exploratory Simulation Study Grounded in Human-AI Reliance Data

**DOI:** 10.2196/99590

**Published:** 2026-08-05

**Authors:** Hiroyuki Tajima

**Affiliations:** 1 Faculty of Nursing, Shumei University, 1-1 Daigaku-cho, Yachiyo, Chiba, 276-0003, Japan, 81 47-488-2111

**Keywords:** clinical decision support, automation bias, large language models, nursing informatics, Monte Carlo simulation, error threshold

## Abstract

**Background:**

Clinical AI decision support is being introduced into nursing practice; however, existing large language models (LLMs) demonstrate only moderate accuracy on complex clinical tasks, raising questions about the level of accuracy required for safe clinical use across varying levels of clinician experience and task complexity.

**Objective:**

The aim of the study is to develop an empirically calibrated simulation model of human-AI reliance and error in nursing decision-making and estimate the AI accuracy required to achieve specified error rate targets.

**Methods:**

A linear reliance model with coefficients for AI accuracy (A), clinician experience (E), and task complexity (C) was calibrated using weighted least squares against 9 empirical data points from 3 independent randomized experiments on AI-assisted decision-making (N=3502). Predicted error was computed as reliance×(1−*A*) across a 27-cell factorial design. Study-level bootstrap (2000 iterations) quantified calibration uncertainty. To contextualize the simulation’s operating range, the accuracy of contemporary general-purpose LLMs on complex clinical tasks was drawn from published benchmarks.

**Results:**

Calibration placed β_A_ at 0.201 (bootstrap 95% CI 0.023‐0.234; *P*(β_A_>0)>.99). For the novice×high-complexity combination, the minimum AI accuracy values required to achieve error rates <10% and<20% were 0.89 and 0.78, respectively (bootstrap 95% CIs 0.88‐0.90 and 0.75‐0.79). At the moderate accuracy levels currently reported for general-purpose LLMs on complex clinical tasks (approximately 0.5‐0.7), the model predicts error rates of roughly 26% to 41% in this high-risk condition.

**Conclusions:**

In this model, keeping predicted error rates below a stringent target (<10%) for high-complexity nursing decision support by novice clinicians requires AI accuracy of at least approximately 0.89, a level that current general-purpose LLMs may not reliably reach on complex clinical tasks. Because the model is calibrated on nonnursing reliance data, these thresholds are illustrative model outputs, not nursing-derived empirical standards. The *A*_threshold_ framework provides a decision-theoretic tool for evaluating the minimum AI accuracy requirement by user-and-task profile. Behavioral validation in nursing contexts remains an essential next step. Because the framework is independent of any specific model, it remains applicable as AI systems improve.

## Introduction

The integration of AI, particularly large language models (LLMs), into nursing education and clinical practice has increased substantially in recent years [1-4]. Although these systems present considerable opportunities to augment clinical reasoning and decision-making, they simultaneously introduce novel risks associated with human reliance on AI-generated outputs [5]. A critical concern is how accurate current AI systems must be for safe use in clinical tasks, particularly because clinical decision support systems can improve care but also introduce safety risks when their outputs are relied upon uncritically [6-8].

Research in human factors and automation science has long demonstrated that users often develop inappropriate levels of trust in automated systems, resulting in overreliance and consequent errors [9,10]. Parasuraman and Riley [9] identified “misuse”—overreliance on automation that produces failures in monitoring or detecting system errors, as a principal failure mode of human-automation interaction. Lee and See [10] subsequently articulated a framework of appropriate reliance in which trust should be calibrated to a system’s demonstrated capabilities. In health care specifically, systematic reviews have documented automation bias as a recurring safety concern in computerized clinical decision support, identifying task complexity, cognitive load, and verification demands as principal mediators of overreliance [11-13]. Despite the established relevance of these frameworks, their quantitative application to health care AI, and to nursing in particular, remains largely underexplored.

Recent empirical studies have begun to characterize human-AI reliance relationships in AI-assisted decision-making. Yin et al [14] conducted 3 preregistered randomized experiments (total n=3793) and reported that participants relied more on AI systems, as the systems’ stated and observed accuracy increased, with agreement fractions rising from approximately 0.75 at 60% accuracy to 0.82 at 95% accuracy in their first experiment. Lu and Yin [15] reported a similar monotonic trend in a subsequent study (n=466, 13,980 trials). In clinical settings, Kücking et al [16] demonstrated that AI recommendation correctness exerted a strong bidirectional influence on the diagnostic accuracy of 223 physicians and nurses assessing wound maceration; correct AI recommendations increased the odds of correct decisions approximately 10-fold (odds ratio [OR] 10.0; *P*<.001), whereas incorrect recommendations significantly reduced accuracy. These findings indicate that automation complacency concerns extend to contemporary AI-based clinical decision support systems and motivate a theoretically grounded, quantitative investigation of the conditions under which AI-assisted clinical errors are most likely to occur.

However, a critical gap remains between the available empirical literature and clinical use decisions. Current studies characterize human-AI reliance in qualitative terms or across a limited range of accuracy levels; yet, they do not provide clinicians or administrators with a decision-theoretic tool for determining whether an AI system, given its measured accuracy, is appropriate for a specific clinical task and user group. Such a tool is increasingly needed because, although LLMs achieve high scores on standardized medical knowledge benchmarks [17], their accuracy on more complex, open-ended clinical decision-making tasks remains only moderate [18,19], placing current systems within an operating range where the clinical safety implications are neither self-evident nor reliably inferable from the raw accuracy value alone.

To address this gap, this study developed a linear simulation model of human-AI reliance in nursing decision-making, empirically calibrated against 9 data points derived from 3 independent randomized experiments on AI-assisted decision-making (total N=3502). Error rates were computed as the product of predicted reliance and AI failure probability and evaluated across a 27-cell factorial design spanning 3 AI accuracy levels, 3 clinician experience levels, and 3 task complexity levels. This work has two primary contributions: (1) empirical calibration of the reliance model from 3 independent large-scale datasets, which avoids the circularity inherent in purely theoretical parameter selection; and (2) introduction of *A*_threshold_, a decision-theoretic parameter that quantifies the minimum AI accuracy required to achieve a specified clinical error rate target for a specific user-and-task profile.

Clinical AI decision support is being introduced into nursing practice without quantitative guidance on the minimum AI accuracy required for safe use. The *A*_threshold_ framework presented here is expected to benefit nursing educators, clinical informaticists, hospital administrators evaluating AI deployment, and regulators developing accuracy standards for clinical AI decision support systems. This work is submitted to the JMIR Nursing theme issue on Artificial Intelligence (AI) in Nursing, addressing the in-scope topics of LLM use in settings where nurses provide care, comparisons of AI algorithm effectiveness in supporting decision-making among nurses, and strengths and limitations of AI technologies in nursing practice.

## Methods

### Study Design

This study used a theoretical Monte Carlo simulation calibrated against published human-AI reliance data. The published human data were used to calibrate the model’s accuracy-related parameters, while all other parameters not determined by calibration were specified a priori from theoretical considerations and tested through sensitivity analysis. To ensure that the simulation’s operating range was ecologically plausible, the accuracy of contemporary general-purpose LLMs on complex clinical tasks was characterized using published benchmarks [18,19] rather than a separate in-house evaluation.

### Ethical Considerations

This study did not involve human participants or the use of patient data; it comprised a computational simulation calibrated against previously published, publicly available aggregate data. Accordingly, institutional review board approval was not required.

### Theoretical Framework and Model Formulation

#### Reliance Model

Human reliance on AI was modeled as a linear function of AI accuracy, clinician experience, and task complexity:

Reliance=β_₀_+β_A_·*A*+β_E_·*E*+β_C_·*C*+β_AE_·(*A*×*E*)+β_AC_·(*A*×*C*)+ε₁(1)

where *A* denotes AI accuracy on [0, 1]; *E* signifies clinical experience normalized by dividing the years of experience by 10 (so that 0, 2, and 10 years map to 0.0, 0.2, and 1.0, respectively); *C* represents task complexity coded as 0.0 (low), 0.5 (moderate), and 1.0 (high); and ε₁~*N*(0, σ=0.05). To preserve its probabilistic interpretation, reliance was clipped to the interval [0, 1].

The linear form was chosen over more elaborate specifications (eg, a logistic trust mediator with a quadratic accuracy penalty) for 3 reasons. First, it is parsimonious, as it contains the minimum parameters needed to capture additive effects of accuracy, experience, and complexity with their 2-way interactions. Second, it can be directly calibrated against published reliance data using standard regression methods, whereas more elaborate specifications add extra parameters that cannot be reliably estimated from the available data. Third, empirical studies of human-AI reliance consistently reported approximately linear relationships between AI accuracy and reliance across the 0.5‐1.0 range [14,15], which empirically justifies the linear specification.

#### Error Model

**Figure 1.**
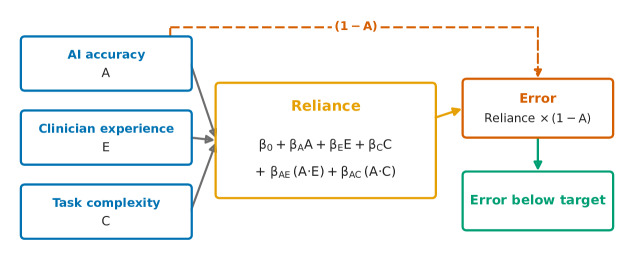
Conceptual model of AI-assisted clinical decision-making. Inputs (AI accuracy A, clinician experience E, and task complexity C) feed the reliance equation with additive main effects and 2-way interactions (*A*×*E* and *A*×*C*). The accuracy-related coefficients β_₀_ and β_A_ were empirically calibrated against 9 data points drawn from 3 independent randomized experiments on AI-assisted decision-making (total N=3502). The remaining coefficients were fixed from prior theoretical considerations. Error is computed as reliance×(1−*A*), so AI accuracy enters the error term both indirectly through reliance and directly through the (1−*A*) factor. The predicted error is then compared against a specified target error rate. β_0_ and β_A_ were calibrated from 9 empirical reliance points [14,15]; β_E_, β_C_, β_AE_, and β_AC_ were fixed from the literature.

Error was modeled as the product of behavioral reliance and the AI failure rate with additive Gaussian noise:

Error=reliance×(1−*A*)+ε₂(2)

where ε₂~*N*(0, σ=0.02), with error subsequently clipped to [0, 1]. This specification assumes that an erroneous AI recommendation leads to a clinical error only to the extent that the clinician relies on it. Figure 1 presents the conceptual model of human-AI interaction in nursing decision-making.

### Model Calibration

The *A*-related coefficients of the reliance model (β_₀_ and β_A_) were calibrated using weighted least squares against 9 empirical data points derived from 3 independent randomized experiments on AI-assisted decision-making. Studies met 3 prespecified criteria: experimental manipulation of AI accuracy at ≥2 levels in the 0.5‐1.0 range, reported behavioral measure of reliance (agreement fraction, switch fraction, or equivalent), and total study n≥100.

In total, 3 studies met all 3 criteria. In all 3 experiments, laypeople recruited via Amazon Mechanical Turk used a machine-learning classifier to predict the outcomes of speed-dating events (whether a given participant would want to meet their date again), and reliance was operationalized as the agreement (or switch) fraction between the participant’s final decision and the model’s prediction. Lu and Yin [15] (experiment 2; n=466 crowdworkers; 13,980 trials) manipulated the designed AI accuracy (50% and 80%) and measured the final agreement rate. Individual-level data were obtained from authors’ publicly available dataset [20] and reanalyzed to obtain worker-level reliance means at each accuracy level (*A*=0.50: n=252; mean 0.642, SD 0.139; *A*=0.80: n=214; mean 0.664, SD 0.134). Yin et al [14] (experiment 1, phase 1; n=1994; stated accuracy ∈ {60%, 70%, 90%, 95%}; agreement fraction with observed accuracy of 80% and experiment 3, phase 2; n=1042; observed accuracy ∈ {55%, 80%, 100%}; agreement fraction averaged across 2 stated accuracy levels) contributed 4 and 3 data points, respectively. Values were extracted from the published figures (Figures 3a and 5a) of Yin et al [14] by visual inspection. The 2 Yin et al [14] experiments used here (experiment 1, phase 1 and experiment 3, phase 2) together comprised 3036 of the 3793 participants reported across that study’s 3 experiments; adding the 466 crowdworkers of Lu and Yin [15] yields the total calibration sample of 3502. Experiments and phases that did not manipulate AI accuracy across at least 2 levels in the 0.5‐1.0 range were not used for calibration.

All calibration targets correspond to the clinician-experience value *E*=0 (laypeople crowdworkers) and the task complexity value *C*=0.5 (moderate complexity; speed-dating outcome prediction requires the integration of multifeature personal profiles). Calibration was performed by minimizing the sum of the squared residuals weighted by the square root of each study’s sample size using the Nelder-Mead simplex algorithm in SciPy (version 1.13; NumFOCUS). The other coefficients (β_E_, β_C_, β_AE_, and β_AC_) were set from prior theoretical considerations and are described in the Parameter Specification section.

Calibration yielded β_₀_=0.471 (baseline reliance at *A*=0, *E*=0, *C*=0.5 with literature-fixed complexity contribution) and β_A_=0.201 (reliance increase per unit of AI accuracy). The calibrated model achieved a root-mean-square error (RMSE) of 0.054 across the 9 empirical points.

To evaluate the precision of the calibrated coefficients given the limited number of independent data sources, study-level bootstrap resampling was run with 2000 iterations. For each iteration, the 3 studies were sampled with replacement, and β_₀_ and β_A_ were recalibrated on the resulting target set. Leave-one-study-out cross-validation was also conducted by excluding each study in turn and recalibrating the remaining 2. The bootstrap distribution and leave-one-out results are presented in the Results section.

### Parameter Specification

The remaining 4 coefficients were specified a priori based on theoretical considerations and were not estimated from the calibration data. The coefficient for clinical experience in the reliance equation (β_E_=−0.20) reflects two complementary mechanisms: (1) expertise development cultivates reliance on internalized clinical knowledge and pattern recognition rather than on external decision aids [21,22] and (2) empirical studies in clinical AI decision support have shown that longer work experience is directly associated with reduced susceptibility to incorrect AI recommendations (OR 1.89 for work experience in a recent study of 223 health care professionals [16]). The coefficient for task complexity (β_C_=+0.20) operationalizes evidence that increased cognitive load is associated with reliance on decision aids [9]. The 2 interaction coefficients (β_AE_=+0.10 and β_AC_=+0.10) represent, respectively, expertise-based attenuation and complexity-based amplification of accuracy effects. These values are theoretically motivated rather than empirically estimated. The specific magnitudes (|β|=0.10‐0.20) were chosen as conservative nominal values, deliberately set at or below the magnitude of the calibrated accuracy slope (|β_A_|≈0.20) so that these unvalidated assumptions would not dominate the empirically calibrated reliance-accuracy relationship; they are not presented as precise estimates. Because 9 aggregate calibration points cannot identify these 4 coefficients empirically, alternative specifications were not selected on the basis of statistical fit; instead, the influence of these values was bounded through the sensitivity analyses described below, which span a plausible range of alternative magnitudes.

### Simulation Conditions

In a Monte Carlo simulation, an outcome of interest is estimated by repeatedly drawing random values from specified probability distributions and averaging the results across many simulated trials; here, this approach propagated the small random variation in the modeled reliance and error terms into stable estimates of the mean predicted error rate for each condition. A full factorial design was used: 3 levels of AI accuracy (0.5, 0.7, and 0.9)×3 levels of clinical experience (0, 2, and 10 years)×3 levels of task complexity (low, moderate, and high)=27 conditions. Each condition was simulated with 10,000 Monte Carlo trials (total n=270,000 simulated decision trials). The pseudorandom number generator was initialized with seed 42 using the numpy default_rng interface for full reproducibility.

### *A*_threshold_ Analysis

The minimum AI accuracy required to achieve a specified clinical error rate target (denoted as *A*_threshold_) was computed analytically for each combination of clinical experience and task complexity. Given the linear reliance specification, the predicted mean error is a quadratic function of *A*:

Error(*A*|*E*, *C*)=[(β_₀_+β_E_·*E*+β_C_·*C*)+(β_A_+β_AE_·*E*+β_AC_·*C*)·*A*]×(1−*A*)(3)

For a specific target error threshold *T*, *A*_threshold_ was computed as the smallest *A* ∈ [0, 1] at which error(*A*|*E*, *C*)≤*T*, obtained through a dense grid search over 1000 values of *A*. In total, 4 target error thresholds were evaluated, namely, 5%, 10%, 15%, and 20%. These values were not adopted from a single validated benchmark; rather, they constitute a graded series of progressively stricter error rate targets intended to span the range of error tolerances plausibly relevant to clinical decision support, from comparatively lenient targets appropriate to low-stakes supportive use to stringent targets appropriate to higher-stakes settings. Consistent with the exploratory aim of this study, the resulting *A*_threshold_ values should be interpreted as normative, hypothesis-generating estimates rather than as empirically validated error rate targets, and the specific threshold relevant to any given deployment will depend on the clinical context.

### Sensitivity Analysis

To assess model robustness, each literature-fixed coefficient (β_E_, β_C_, β_AE_, and β_AC_) was systematically perturbed by ±20% from its baseline value. The calibrated coefficients (β_₀_ and β_A_) were not perturbed because they were empirically determined rather than theoretically assumed. For each variant, the entire 27-cell simulation was rerun, and the resulting cell-level error rates were compared with baseline using Spearman rank correlation and maximum absolute error difference.

### Joint Human-AI Decision Model

The primary error specification (error=reliance×(1−*A*)) attributes a clinical error solely to reliance on an incorrect AI recommendation and therefore represents the limiting case, in which the clinician contributes no independent accuracy. Human-AI teams do not always outperform either party alone and can even perform worse on decision-making tasks [23,24]—for example, a randomized trial found that giving physicians access to an LLM did not significantly improve their diagnostic reasoning over conventional resources [25]. In addition, users frequently overrely on AI by following incorrect recommendations [13], and appropriate reliance requires accepting correct advice while rejecting incorrect advice [26]. For these reasons, a joint human-AI decision model was additionally analyzed that represents both outcomes of an AI-assisted decision explicitly: when the clinician follows the AI, an error occurs if the AI is incorrect; and when the clinician overrides the AI, an error occurs if the clinician’s own independent judgment is incorrect. Writing *R* for reliance (the probability of following the AI), *A* for AI accuracy, and *p*_c_ for the clinician’s independent accuracy, the expected team error is error_joint_=*R*×(1−*A*)+(1−*R*)×(1−*p*_c_). The primary model is recovered exactly as the special case *p*_c_→1 (a clinician who is always correct when acting independently); this was verified to reproduce the reported *A*_threshold_ values (0.894 and 0.779 for the novice×high-complexity cell at the <10% and <20% targets). An asymmetric variant additionally distinguished passive deference to an incorrect recommendation from active correction of one by scaling the corrective term by a factor ρ ∈ [0, 1], where ρ=1 recovers the symmetric model, and smaller ρ represents clinicians who more often catch and overturn a wrong recommendation. *A*_threshold_ was recomputed under this model across clinician-accuracy levels *p*_c_ ∈ {0.6, 0.7, 0.8, 0.9, 1.0}.

### Extended Sensitivity Analysis

To probe robustness beyond the ±20% perturbations, 3 additional analyses were performed. First, each literature-fixed coefficient was varied over a wide one-at-a-time range (−100%, −50%, +50%, and +100% of its baseline magnitude, together with a full sign reversal), and the 27-cell simulation was rerun for each variant. Second, a global Monte Carlo analysis simultaneously sampled all 4 literature-fixed coefficients from prior distributions across 50,000 draws under 2 priors: a theory-signed prior that preserved the assumed direction of each coefficient while allowing its magnitude to vary widely, and a fully sign-agnostic prior that additionally permitted every assumed sign to reverse. The resulting distribution of *A*_threshold_ values for the highest-risk (novice×high-complexity) and lowest-deference (experienced×low-complexity) cells was summarized by its median, 5th- to 95th-percentile interval, and the probability of exceeding an accuracy of 0.70. Third, to test whether the conclusions were an artifact of the linear reliance form, 2 bounded alternative structures—a logistic (sigmoid) reliance function and a concave saturating function—were fit to the same 9 calibration points, and *A*_threshold_ was recomputed from each. These analyses are reported in the Results section, summarized in Multimedia Appendix 1, and provided in full in the repository.

## Results

### Model Calibration

Weighted least-squares calibration of the reliance model against the 9 empirical data points yielded β_₀_=0.471 and β_A_=0.201. The calibrated model achieved an RMSE of 0.054 across the calibration targets (unweighted *R*^2^=0.34, reflecting between-study heterogeneity in baseline reliance that the model does not attempt to capture). Multimedia Appendix 2 overlays the calibrated model predictions on the 9 empirical data points, stratified by the source study.

Study-level bootstrap resampling (2000 iterations) yielded a 95% CI for β_A_ of 0.023‐0.234, which reflects the small number of independent calibration sources and the between-study heterogeneity in baseline reliance. Despite this width, the sign of β_A_ was stable across all 2000 bootstrap iterations (*P*(β_A_>0)>.99), confirming that the monotonic positive relationship between AI accuracy and reliance is empirically robust. The leave-one-study-out cross-validation produced recalibrated β_A_ values of 0.094 (excluding Lu and Yin [15]), 0.246 (excluding experiment 3 by Yin et al [14]), and 0.199 (excluding experiment 1 phase 1 by Yin et al [14]). This finding indicates that the calibration depends most heavily on the study by Lu and Yin [15] but does not collapse upon its removal. Per-study calibration yielded β_A_ estimates of 0.023, 0.037, and 0.158, all positive: the joint calibration value of 0.201 represents the precision-weighted aggregate across these heterogeneous study-level estimates.

Two features of the calibration result warrant emphasis. First, the calibrated slope (β_A_=0.201) indicates that each 0.10-unit increase in AI accuracy corresponds to approximately a 0.02-unit increase in the predicted mean reliance. This is considerably weaker than the reliance-accuracy relationships assumed in some theoretically motivated frameworks and reflects the fact that human reliance on AI systems is sensitive to, but not dominated by, the accuracy of the system. Second, the 3 studies contributing calibration data have different baseline reliance levels (Lu and Yin [15]: mean≈0.65; experiment 1 by Yin et al [14]: mean≈0.78; and experiment 3 by Yin et al [14]: mean≈0.82), a heterogeneity that the linear model averages across rather than resolves. Residuals were accordingly approximately symmetric around 0, but the RMSE was markedly larger than the SEs of the study means, an outcome we interpret as reflecting unmodeled study-level factors rather than misspecification of the functional form in the accuracy dimension.

### Predicted Reliance and Error Rates

Table 1 presents the predicted error rates across all 27 simulation conditions. The underlying predicted reliance values ranged from 0.421 (experienced clinicians on low-complexity tasks with *A*=0.5) to 0.938 (novice clinicians on high-complexity tasks with *A*=0.9), reflecting the combined influence of accuracy, experience, and complexity on the tendency to accept AI recommendations. Notably, the calibrated slope on *A* is sufficient to produce an approximately 0.08-unit increase in mean reliance between *A*=0.5 and *A*=0.9, but insufficient to generate an interior maximum of predicted error rate as a function of *A*.

**Table 1.** Predicted error rates by simulation condition^a^.

AI accuracy	Experience (years)	Low complexity, mean (SD)	Moderate complexity, mean (SD)	High complexity, mean (SD)
0.5	0	0.29 (0.03)	0.35 (0.03)	0.41 (0.03)
0.5	2	0.27 (0.03)	0.33 (0.03)	0.40 (0.03)
0.5	10	0.21 (0.03)	0.27 (0.03)	0.34 (0.03)
0.7	0	0.18 (0.03)	0.22 (0.03)	0.26 (0.03)
0.7	2	0.18 (0.03)	0.22 (0.03)	0.26 (0.03)
0.7	10	0.14 (0.03)	0.19 (0.03)	0.23 (0.03)
0.9	0	0.07 (0.02)	0.08 (0.02)	0.09 (0.02)
0.9	2	0.06 (0.02)	0.08 (0.02)	0.09 (0.02)
0.9	10	0.05 (0.02)	0.07 (0.02)	0.08 (0.02)

a Values are mean (SD) of simulated error rates across 10,000 Monte Carlo trials per cell (seed=42).

**Figure 2.**
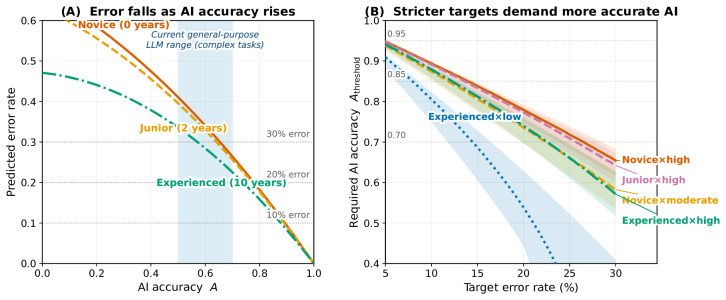
Predicted error rates and accuracy thresholds for AI decision support in nursing. (A) Predicted mean error rate as a function of AI accuracy *A* at high task complexity (*C*=1.0) for 3 experience levels. The horizontal dotted lines mark 10%, 20%, and 30% target error rates. The shaded vertical band marks a reference range for the accuracy of contemporary general-purpose LLMs on complex clinical tasks (approximately 0.5‐0.7), based on published benchmarks [18,19]. (B) *A*_threshold_ curves plotting the required AI accuracy against the target error-rate threshold for 5 representative experience-by-complexity combinations. Solid and dashed lines show point estimates from the calibrated model. The shaded bands represent study-level bootstrap 95% CIs (2000 iterations). Horizontal reference lines mark a reference LLM accuracy level (0.70), plausible near-term frontier LLM accuracy (0.85), and demanding target accuracy (0.95). Note that *A*_threshold_ uncertainty is narrow at low target error rates (left side) and wider at higher target error rates (right side), reflecting the mathematical structure of error=reliance×(1−*A*). LLM: large language model.

The predicted error rates exhibited a clean 3-tier structure when grouped by AI accuracy level. At *A*=0.9, all 9 cells fell below a 10% error rate (range 0.054‐0.094). At *A*=0.7, the predicted error rates ranged from 0.145 to 0.265, spanning the 10%‐20% and 20%‐30% ranges, with all 3 novice cells exceeding 18% predicted error. At *A*=0.5, the predicted error rates ranged from 0.211 to 0.411, with all 6 moderate- and high-complexity cells exceeding 27% predicted error. For the cell most representative of the clinically high-risk scenario (novice clinicians performing high-complexity tasks), the predicted error rates were 0.411 at *A*=0.5, 0.265 at *A*=0.7, and 0.094 at *A*=0.9 (Figure 2A).

The *A*=0.7 simulation condition is closest to the accuracy levels currently reported for general-purpose LLMs on complex clinical tasks [18,19], in which the predicted error rates for novice clinicians range from 0.184 (low-complexity tasks) to 0.265 (high-complexity tasks). At high task complexity, the condition most relevant to ambiguous clinical presentations, the predicted error rate of 0.265 implies that approximately 1 in 4 AI-supported clinical decisions would be incorrect when the system is used by a novice clinician at this accuracy level.

### *A*_threshold_ Analysis

Table 2 presents *A*_threshold_ values, the minimum AI accuracy required to achieve a specified error rate, for each of the 9 experience-by-complexity combinations at 4 target error thresholds (5%, 10%, 15%, and 20%). For novice clinicians performing high-complexity tasks, the minimum AI accuracy values required to achieve error rates <10%, <15%, and <20% were 0.894, 0.838, and 0.779, respectively. Corresponding values for experienced clinicians performing low-complexity tasks were 0.806, 0.686, and 0.539, respectively. These differences highlight the interaction between clinician experience, task complexity, and the minimum accuracy requirement for clinical use. Because the reliance model is calibrated on nonnursing behavioral data, these numerical thresholds should be read as illustrative outputs of the model rather than as nursing-derived empirical standards; their value lies in the relative ordering across user-and-task profiles rather than in the specific accuracy figures.

**Table 2.** *A*_threshold_ values—minimum AI accuracy required to achieve specified error rate targets^a^.

Experience and complexity	Error<5%	Error<10%	Error<15%	Error<20%
0 years				
Low	0.925	0.845	0.759	0.670
Moderate	0.939	0.874	0.807	0.736
High	0.949	0.894	0.838	0.779
2 years				
Low	0.922	0.839	0.749	0.653
Moderate	0.937	0.870	0.800	0.726
High	0.947	0.891	0.834	0.773
10 years				
Low	0.909	0.806	0.686	0.539
Moderate	0.929	0.851	0.766	0.670
High	0.942	0.879	0.812	0.740

a *A*_threshold_ is the smallest AI accuracy value *A* ∈ [0, 1] at which the calibrated model’s predicted mean error rate falls below the specified error rate target, computed analytically from the closed-form quadratic expression derived from the linear reliance specification. Values are rounded to 3 decimal places and were computed from the full-precision calibrated coefficients; exact coefficient values are available in the repository.

Bootstrap propagation of calibration uncertainty into *A*_threshold_ values revealed an important asymmetry: although β_A_ has a wide bootstrap 95% CI, the *A*_threshold_ values are tightly bounded. For the novice×high-complexity combination, the bootstrap 95% CIs for the *A*_threshold_ to achieve error rates of 10%, 15%, and 20% were 0.877‐0.898, 0.814‐0.845, and 0.750‐0.792, respectively. This tight propagation reflects the mathematical structure of error=reliance×(1−*A*): at low target error rates, the (1−*A*) factor must be small, which constrains *A* to lie close to 1 regardless of the exact value of β_A_ within its CI. Correspondingly, across the accuracy range currently reported for general-purpose LLMs on complex clinical tasks [18,19], the predicted error rate for novice clinicians on high-complexity tasks ranges from approximately 0.26 at the upper end of that range to approximately 0.41 at the lower end. The conclusion that AI systems operating in this accuracy range yield error rates well above stringent targets for this user-and-task combination is robust across the bootstrap distribution of the calibrated coefficients.

Figure 2B presents the *A*_threshold_ function graphically, plotting the required AI accuracy against the target error-rate threshold for 5 representative experience-by-complexity combinations. Horizontal reference lines mark a reference LLM accuracy level (0.70), plausible near-term frontier LLM accuracy (0.85), and demanding target accuracy (0.95). At a reference accuracy level of 0.70, a total of 4 of the 9 cells achieve predicted error rates <20%: the 3 low-complexity cells (ranging from 14.5% for experienced clinicians to 18.4% for novices) and the experienced×moderate-complexity cell (18.5%). The remaining 5 cells—comprising novice and 2-year-experience clinicians on moderate- or high-complexity tasks, plus experienced clinicians on high-complexity tasks—have predicted error rates ranging from 21.6% to 26.5%, consistently exceeding a 20% error rate.

### Sensitivity Analysis

When each of the 4 literature-fixed coefficients (β_E_, β_C_, β_AE_, and β_AC_) was varied by ±20% from its baseline value, the overall error ranking of the 27 cells was nearly preserved in every perturbation. Across all 8 perturbations (4 coefficients×2 directions), the Spearman rank correlation between the perturbed and baseline cell-level error rankings ranged from 0.991 to 1.000, with a mean of 0.998 (SD 0.003). The maximum absolute change in any cell’s predicted error rate across all perturbations was 0.020, occurring for the β_E_+20% perturbation in the experienced×moderate-complexity×*A*=0.5 cell (baseline 0.273; perturbed 0.253). These results indicate that the principal conclusions of the simulation—the tiered error-rate structure of the 27 cells and the *A*_threshold_ values reported in Table 2—are robust to moderate perturbation of the literature-fixed coefficients.

### Joint Decision Model and Extended Robustness

Under the joint human-AI decision model, the principal conclusion for the highest-risk condition was unchanged. For novice clinicians performing high-complexity tasks, the minimum AI accuracy required to keep predicted team error below 10% moved only from 0.894 at *p*_c_→1 to 0.917 at *p*_c_=0.6, and the threshold for the<20% target moved from 0.779 to 0.818 across the same range; because reliance is high in this cell, the override path contributes little, and clinician accuracy has limited leverage. A qualitatively different pattern emerged in the lowest-deference cell (experienced clinicians on low-complexity tasks), where team error acquired an irreducible floor equal to (1−*R*(*A*=1))×(1−*p*_c_). This floor grew from 0% at *p*_c_→1 to 8.6% at *p*_c_=0.8 and 17.1% at *p*_c_=0.6, so that the<10% target became unreachable by any AI accuracy once *p*_c_ fell to 0.7 or below. This structural feature—that improving AI accuracy alone cannot drive team error below the clinician’s own error contribution—indicates that a single accuracy number is insufficient and that the human contribution to the joint decision must be represented. Under the asymmetric variant, allowing clinicians to correct a wrong recommendation (ρ<1) lowered the required accuracy substantially (for the novice×high-complexity cell at the <20% target, from 0.779 at ρ=1 to 0.673 at ρ=0.7 and 0.516 at ρ=0.5), indicating that the primary model is conservative with respect to correction.

The extended sensitivity analyses reinforced the ±20% results. Under wide one-at-a-time perturbation (up to ±100% and sign reversal of each literature-fixed coefficient), the novice×high-complexity threshold was mathematically invariant to β_E_ and β_A_ (both multiply the experience term, which is 0 in that cell) and varied only with the complexity-related coefficients; even under a full sign reversal of β_C_, the required accuracy for the <10% target remained between 0.805 and 0.900, and the rank ordering of the 27 cells was preserved (Spearman ρ≥0.77 across all perturbations, and ρ>0.95 for most). In the global Monte Carlo analysis (50,000 draws per prior), the theory-signed prior yielded a novice×high-complexity *A*_threshold_ with median 0.894 (5th-95th percentile 0.863‐0.900) for the <10% target and 0.780 (5th-95th percentile 0.709‐0.800) for the <20% target, with the required accuracy exceeding 0.70 in 100% and 97.4% of draws, respectively. Even under the fully sign-agnostic prior, in which every assumed coefficient sign was permitted to reverse, the <10% threshold for this cell retained a median of 0.844 and exceeded 0.70 in 84.4% of the sampled parameter space; values in the low tail arose only under sign combinations contrary to the theoretical expectations. Finally, both bounded alternative reliance structures fit the 9 calibration points essentially as well as the linear form (RMSE 0.0542 for the logistic and concave-saturating forms vs 0.054 for the linear form) and produced near-identical thresholds for the novice×high-complexity cell (0.900 and 0.794‐0.795 for the <10% and <20% targets, respectively), indicating that the conclusions are not an artifact of the linear specification. Multimedia Appendix 1 summarizes the joint-model and global-sensitivity results.

## Discussion

### Principal Findings

In this study, an empirically calibrated simulation model was developed to quantify the AI accuracy required to achieve specified error rate targets in nursing decision-making across combinations of clinician experience and task complexity. Three principal findings warrant emphasis. We emphasize at the outset that the contribution of this work is a reusable, model-agnostic theoretical framework rather than an evaluation of any particular AI system. Its value lies in the mapping it defines from AI accuracy, clinician experience, and task complexity to a predicted error rate and a corresponding minimum-accuracy requirement—a mapping that does not depend on which model is current or on the specific accuracy figure entered into it.

First, the moderate accuracy levels currently reported for general-purpose LLMs on complex clinical tasks [18,19] fall within an operating zone where the predicted error rates exceed stringent targets for novice clinicians performing complex tasks. At the upper end of this reported range (approximately 0.70), the simulation predicts an error rate of approximately 0.26 in the novice×high-complexity cell—rising to roughly 0.41 at the lower end of the range—substantially exceeding the stringent error targets relevant to autonomous clinical decision support in high-risk clinical scenarios.

Second, the calibrated reliance model—grounded in 9 empirical data points from 3 independent randomized experiments (total N=3502)—indicates that human reliance on AI recommendations rises monotonically with AI accuracy but at a modest rate (β_A_=0.20). This slope is considerably shallower than the reliance-accuracy relationships assumed in some theoretical frameworks and reflects the cumulative evidence that users calibrate their reliance in response to AI accuracy, but do so imperfectly and with substantial residual reliance even on low-accuracy systems. Because reliance does not rise steeply enough to offset the declining AI failure probability at high accuracy levels, the predicted clinical error rates in this model decrease monotonically as AI accuracy increases, without an interior maximum. The clinical implication is that no “dangerous middle” region exists in which error rates peak; the clinical concern, rather, is the absolute magnitude of error rates across a broad low-to-moderate accuracy range.

Third, *A*_threshold_ values, the minimum AI accuracy required to achieve a specified error rate target, exhibit a pronounced dependence on clinician experience and task complexity. For the highest-risk combination (novice clinicians performing high-complexity tasks), achieving an error rate <10% requires AI accuracy of at least 0.89, and achieving an error rate <20% requires accuracy of at least 0.78. For experienced clinicians performing low-complexity tasks, the corresponding thresholds are 0.81 and 0.54. These differences, spanning up to 24 percentage points of the required accuracy across user-and-task profiles (at the <20% error target), indicate that a single accuracy benchmark cannot meaningfully govern clinical use decisions; the threshold depends on the user-and-task context.

### Comparison With Prior Work

The predictions of the proposed model converge with findings from 2 independent lines of empirical research on human-AI interaction.

First, in the AI-assisted decision-making literature, the calibrated model is grounded directly in the reliance data from Lu and Yin [15] and Yin et al [14], representing 3 randomized experiments with a combined sample of 3502 participants. The linear form of the model aligns with the empirically observed monotonic relationship between AI accuracy and human reliance, and the absence of an interior reliance maximum in the range *A* ∈ [0.5, 1.0], observed in both studies, directly shaped the structural form of the reliance equation. This empirical basis distinguishes the present framework from earlier theoretical treatments that posited nonlinear reliance patterns without supporting behavioral data.

Second, in the clinical decision support literature, Kücking et al [16] reported that AI recommendation correctness exerted a strong bidirectional influence on the diagnostic performance of 223 physicians and nurses (OR 10.0; *P*<.001 for correct AI recommendations; reciprocal declines for incorrect recommendations). They also found that longer work experience was associated with higher diagnostic accuracy (OR 1.89), and formal qualifications further improved accuracy (OR 1.40). These clinical expertise effects are directionally consistent with the negative experience coefficient (β_E_=−0.20) specified a priori in the present model. In a related study by the same group [27], the overall human diagnostic accuracy averaged 79.3% (up to 85% in the formally qualified subgroup); by comparison, general-purpose LLMs have been reported to achieve lower accuracy on complex clinical tasks [18,19], situating the operating range explored in the present simulation within a clinically meaningful band relative to human performance.

The introduced *A*_threshold_ framework is best understood in relation to 3 established frameworks for human-automation interaction. Lee and See [10] proposed a process-oriented framework in which trust calibration determines appropriate reliance but did not provide quantitative guidance for translating system accuracy into use case decisions. Parasuraman and Riley [9] articulated a use/misuse/disuse/abuse taxonomy that classifies human-automation interaction failure modes but treats accuracy as an input among many rather than as a use-determining quantity. More recently, the literature on trustworthy AI [6] has emphasized explainability, fairness, and accountability as design principles, but has generally not specified accuracy thresholds for clinical use.

The present framework differs from prior approaches in 3 ways. First, it converts a single quantitative input—AI accuracy on the intended task—into a deployment decision using an empirically calibrated function. Second, it explicitly adjusts the deployment threshold based on user experience and task complexity, recognizing that no universal accuracy cutoff is appropriate across different use cases. Third, the graphical guidance provided in Figure 2B allows clinicians and administrators to read off the minimum required accuracy for a specific error tolerance and user-task profile without requiring statistical expertise. These properties make the *A*_threshold_ framework complementary to, rather than competitive with, existing process- and design-oriented frameworks: *A*_threshold_ provides a quantitative gating criterion that can be applied alongside trust calibration and trustworthy AI principles in deployment review processes.

Taken together, the present framework is consistent with both the quantitative reliance patterns observed in general-purpose AI-assisted decision-making and the qualitative expertise effects observed in clinical decision support. The principal gap in the empirical foundation—behavioral reliance data collected specifically in nursing populations performing clinical decision-making tasks across multiple AI accuracy levels—remains a priority for future research.

### Theoretical Integration

The finding that *A*_threshold_ varies strongly with task complexity can be interpreted through the lens of dual-process theory [28]. At low task complexity, system 2 (analytical) processing may remain engaged even when AI accuracy is moderate, because the clinician retains sufficient cognitive resources to evaluate the AI recommendation independently. At high task complexity, by contrast, system 1 (automatic) processing may predominate, as cognitive resources are preferentially allocated to the task itself rather than to critical evaluation of the AI recommendation. This asymmetry provides a theoretical rationale for the model’s larger complexity-by-accuracy interaction coefficient and for the model-implied result that novice clinicians, who have not yet developed efficient system 2 strategies for clinical reasoning [21,22], face the most stringent AI accuracy requirements.

Within the framework of Lee and See [10], the model implies that appropriate reliance is unlikely to be fully achieved through user education or interface design alone when AI accuracy falls substantially below the *A*_threshold_ for the relevant user-and-task combination. In such contexts, the residual error rate is a function of system accuracy rather than of user awareness, and the appropriate intervention is either to restrict AI use to user-and-task combinations for which the *A*_threshold_ is met or to improve AI accuracy itself.

A further implication concerns the durability of the framework, as language models continue to improve. Because the *A*_threshold_ framework maps any given AI accuracy onto a predicted error rate and a corresponding minimum-accuracy requirement, its utility does not depend on the accuracy level of any particular contemporary model. As model performance advances, the same framework can be reapplied to locate an improved system within the accuracy-error landscape and to re-evaluate which user-and-task combinations it can safely support. The specific operating range attributed here to current general-purpose LLMs is thus a movable reference point within a framework that is itself invariant to such change.

### Clinical Implications

The following implications are exploratory and contingent on behavioral validation in nursing settings; they describe how the *A*_threshold_ framework might inform practice rather than offering validated recommendations and should be interpreted in conjunction with the limitations. In particular, a system’s meeting a given *A*_threshold_ on a benchmark should be treated as a necessary but not a sufficient condition for nursing use, and the framework must not be interpreted as authorization to deploy a system without behavioral validation in the relevant nursing context.

First, the minimum AI accuracy required for clinical use should be specified as a function of the intended user and task, not as a single benchmark. Although an AI system operating at the moderate accuracy levels typical of current general-purpose LLMs on complex clinical tasks [18,19] may be suitable for use by experienced clinicians in low-complexity tasks, it is predicted to produce error rates >25% when used by novice clinicians in high-complexity decision-making. Therefore, clinical use decisions should be based on the specific *A*_threshold_ value that corresponds to the intended user population and task profile, rather than on the raw AI accuracy value alone. Moreover, the tolerable error rate is itself context-dependent—shaped by the clinical setting, the severity of potential harm, the degree of human supervision, and whether the system is used in a supportive or an autonomous capacity—so a target that is reasonable for an educational support tool may be inadequate for high-risk acute care.

Second, as AI is increasingly integrated across nursing practice and curricular reform has been called for to prepare nurses to work safely alongside these technologies [29], nursing education programs should incorporate explicit instruction on the relationship among AI accuracy, user experience, and clinical error rates. Students should understand that AI systems in the low-to-moderate accuracy range typical of current general-purpose LLMs may be inadequate for autonomous use in high-complexity clinical decisions. In these settings, AI should augment, not replace, clinical judgment. The *A*_threshold_ framework offers a practical decision tool for this educational purpose.

Third, clinical workflows could adopt differentiated deployment protocols informed by the *A*_threshold_ framework. When the current AI accuracy for user-and-task combinations falls below the relevant *A*_threshold_, enhanced monitoring safeguards, such as mandatory verification steps, second-clinician review, or confidence-based routing, should be applied preferentially. These measures provide a practical intermediate solution while AI accuracy continues to increase toward levels suitable for autonomous use.

Fourth, regulatory and institutional review processes for clinical AI decision support could be informed by encouraging deployment proposals to specify the intended user population and task profile and to consider the corresponding *A*_threshold_. Current regulatory frameworks do not typically request this level of user-and-task-specific justification; the *A*_threshold_ framework is offered as a candidate quantitative input to such review, pending behavioral validation, rather than as a validated regulatory standard. This need is underscored by recent calls for explicit regulatory oversight of LLMs used in health care [30,31].

### Limitations

Several limitations merit consideration.

First, although the accuracy-related coefficients of the reliance model were empirically calibrated against published behavioral data, the framework itself remains a theoretical construct, and its predictions require direct behavioral validation. Calibration confirms that the monotonic reliance slope is consistent with empirical observations in AI-assisted decision-making, but it does not establish that the noncalibrated coefficients (β_E_, β_C_, β_AE_, and β_AC_) accurately reflect nurses performing clinical tasks. The same caution applies to the joint decision model introduced in response to review: the clinician’s independent accuracy (*p*_c_) and the correction factor (ρ) are illustrative parameters rather than calibrated quantities, so the joint-model results should be read as a structured sensitivity analysis over a plausible range of clinician behavior—showing which conclusions are robust to, and which depend on, the human contribution to the decision—rather than as point predictions for any specific clinician accuracy. The framework should therefore be interpreted as a decision-support and hypothesis-generating tool rather than as a validated predictive model for individual clinical use.

Second, the accuracy levels used to contextualize the simulation’s operating range were drawn from published benchmarks of general-purpose LLMs on complex clinical tasks rather than from a nursing-specific evaluation. Reported LLM accuracy varies substantially across models, prompting strategies, task types, and clinical domains, and the accuracy of frontier LLMs on complex clinical tasks remains an active area of research. The operating range examined here should therefore be regarded as an illustrative band informed by the current literature rather than as a definitive estimate of any specific system’s clinical performance.

Third, calibration data were drawn from 3 studies that differed systematically in baseline reliance levels (Lu and Yin [15]: ~0.65; experiment 1 by Yin et al [14]: ~0.78; and experiment 3 by Yin et al [14]: ~0.82). The linear model averages across this heterogeneity, producing an RMSE (0.054) larger than the SEs of individual study means. Therefore, the calibrated slope (β_A_=0.201) should be interpreted as a central tendency across heterogeneous contexts rather than a precise parameter applicable to any specific setting. Part of this heterogeneity may reflect methodological differences in how AI accuracy was manipulated (stated, observed, or designed), which the calibration pooled as proxies for the underlying *A* variable. The heterogeneity is informative and motivates future research parameterizing contextual factors beyond AI accuracy. With only 3 independent calibration studies, the study-level bootstrap has a limited sample space, so the reported CIs should be viewed as lower bounds on the true uncertainty. Consistent with this, the *A*_threshold_ values are reported to 3 decimal places to reflect the numerical precision of the simulation, not the empirical precision of the underlying parameter, which is bounded by a wide CI whose lower limit approaches 0. The thresholds are therefore best interpreted in relative and ordinal terms—comparing the accuracy demands of different user-and-task profiles—rather than as exact accuracy targets to be applied at the reported number of significant figures.

Fourth, the calibration data were derived from studies in which crowdworkers predicted speed-dating outcomes rather than clinicians making clinical decisions. Although this is the most empirically rich reliance-by-accuracy source currently available, the clinical context gap is substantial. Clinicians may exhibit baseline reliance, accuracy sensitivities, and experience effects that differ from those of crowdworkers, and the task complexity assigned to speed-dating prediction (*C*=0.5) reflects a qualitative judgment rather than direct measurement. Even so, the directional structure of the model is consistent with recent experimental evidence from clinical populations that include nurses. In a web-based experiment with 223 dermatologists, reliance on AI was stronger for correct than for incorrect advice and decreased with greater medical experience [32]. More directly, in a wound-maceration study, 223 physicians and nurses made decisions that were sensitive to whether the AI recommendation was correct or incorrect [16], and a related report measuring the rate of agreement with incorrect recommendations found that higher diagnostic performance and certified wound-care training were associated with lower agreement [33]. These patterns match the positive accuracy-reliance slope and the experience-based attenuation encoded in the model, supporting cautious extrapolation of its directional structure to clinical settings. Nursing-specific calibration data obtained across multiple AI accuracy levels would substantially strengthen the empirical foundation of the framework.

Fifth, the linear reliance specification was chosen for parsimony and tractability, since 9 aggregate calibration points are insufficient to identify more elaborate functional forms. Alternative specifications (logistic, power-law, and segmented-linear) might provide superior fit with richer data. The linear form should therefore be interpreted as the simplest specification consistent with the available data, not as a claim that the underlying reliance-accuracy relationship is truly linear.

Sixth, the model does not incorporate contextual factors—such as time pressure, team dynamics, organizational accountability, and environmental stressors—that are known to influence clinical decision-making. These factors likely contribute substantially to real-world reliance and to the observed between-study heterogeneity. Future investigations should explicitly parameterize the most influential contextual factors, which will require more calibration data than are currently available.

Seventh, the reliance and error expressions are clipped to [0, 1] to preserve their probabilistic interpretation. Within the reported 27-cell grid, clipping affected <1% of simulated values and did not materially alter the reported results. At extreme accuracy levels outside the reported grid, clipping becomes more frequent, and the linear reliance expression should not be extrapolated beyond its reported domain.

### Conclusions

This study provides an empirically calibrated simulation framework for quantifying the AI accuracy required to achieve specified error rate targets in nursing decision-making. Calibration against 9 empirical data points derived from 3 independent randomized experiments (total N=3502) indicates that human reliance on AI rises gradually but modestly with AI accuracy. For the highest-risk combination—novice clinicians performing high-complexity tasks—the predicted clinical error rates remain above a stringent target (eg, <10%) when AI accuracy is below approximately 0.89. The moderate accuracy levels currently reported for general-purpose LLMs on complex clinical tasks [18,19] fall well below this threshold, suggesting that, on present evidence, such systems are not yet adequate for autonomous use by novice nurses on complex clinical decisions and should serve as an adjunct to, rather than a replacement for, clinical judgment. The *A*_threshold_ framework introduced here provides a decision-theoretic tool for specifying minimum accuracy requirements as a function of clinician experience and task complexity, and it can inform AI deployment, nursing education, and regulatory policy. Direct behavioral validation in nursing populations remains an essential next step.

## Supplementary material

10.2196/99590 Multimedia Appendix 1 Extended robustness analyses. (A) Minimum AI accuracy (*A*_threshold_) required to keep predicted team error below the 10% and 20% targets under the joint human-AI decision model, plotted against the clinician’s independent accuracy *p*_c_, for the highest-risk (novice×high-complexity) and lowest-deference (experienced×low-complexity) cells. The primary model corresponds to *p*_c_→1. In the lowest-deference cell, the team error acquires an irreducible floor that grows as *p*_c_ declines, so the 10% target becomes unreachable by any AI accuracy at *p*_c_≤0.7. (B) Distribution of the novice×high-complexity *A*_threshold_ from the global Monte Carlo sensitivity analysis (50,000 draws per prior) for the <10% and <20% targets, under a theory-signed prior and a fully sign-agnostic prior. The vertical line marks the reported estimate (0.894) and the dotted line the 0.70 anchor.

10.2196/99590 Multimedia Appendix 2 Calibration of the linear reliance model against 9 empirical data points from 3 independent studies (N=3502).

## Abbreviations

LLM: large language model
OR: odds ratio
RMSE: root-mean-square error
